# Supplementary material for: Multi‐dataset identification of innovative feature genes and molecular mechanisms in keratoconus
Source: J Cell Mol Med. 2024 Sep 19;28(18):e70079. doi: 10.1111/jcmm.70079 (PMC11412914; doi:10.1111/jcmm.70079)
Supplement: Supplementary file 2 — Material S2. [file JCMM-28-e70079-s002.zip › 7.GSEA analysis/5.RNF217-AS1/KEGG_AMINOACYL_TRNA_BIOSYNTHESIS.html]

Details for gene set KEGG\_AMINOACYL\_TRNA\_BIOSYNTHESIS[GSEA]

|  || Dataset | RNF217-AS1.group.cls#h\_versus\_l |
| Phenotype | group.cls#h\_versus\_l |
| Upregulated in class | l |
| GeneSet | KEGG\_AMINOACYL\_TRNA\_BIOSYNTHESIS |
| Enrichment Score (ES) | -0.49406824 |
| Normalized Enrichment Score (NES) | -1.4912866 |
| Nominal p-value | 0.042424243 |
| FDR q-value | 0.32084894 |
| FWER p-Value | 0.772 |
Table: GSEA Results Summary

  

Fig 1: Enrichment plot: KEGG\_AMINOACYL\_TRNA\_BIOSYNTHESIS      
 Profile of the Running ES Score & Positions of GeneSet Members on the Rank Ordered List

  

| SYMBOL | TITLE | RANK IN GENE LIST | RANK METRIC SCORE | RUNNING ES | CORE ENRICHMENT || 1 | VARS2 | na | 11011 | 0.070 | -0.2719 | No |
| 2 | WARS2 | na | 14296 | 0.027 | -0.3489 | No |
| 3 | CARS2 | na | 14678 | 0.023 | -0.3451 | No |
| 4 | TARS2 | na | 15215 | 0.017 | -0.3496 | No |
| 5 | SEPSECS | na | 15692 | 0.012 | -0.3557 | No |
| 6 | YARS2 | na | 18238 | -0.013 | -0.4206 | No |
| 7 | RARS2 | na | 19567 | -0.026 | -0.4421 | No |
| 8 | NARS2 | na | 21372 | -0.044 | -0.4656 | Yes |
| 9 | EARS2 | na | 21410 | -0.044 | -0.4379 | Yes |
| 10 | PARS2 | na | 21483 | -0.045 | -0.4107 | Yes |
| 11 | FARSB | na | 22140 | -0.052 | -0.3956 | Yes |
| 12 | LARS2 | na | 22786 | -0.059 | -0.3756 | Yes |
| 13 | IARS2 | na | 22874 | -0.060 | -0.3390 | Yes |
| 14 | AARS2 | na | 23367 | -0.066 | -0.3100 | Yes |
| 15 | HARS2 | na | 23575 | -0.069 | -0.2708 | Yes |
| 16 | FARSA | na | 24375 | -0.081 | -0.2414 | Yes |
| 17 | SARS2 | na | 24810 | -0.087 | -0.1972 | Yes |
| 18 | FARS2 | na | 24825 | -0.088 | -0.1407 | Yes |
| 19 | MTFMT | na | 25200 | -0.094 | -0.0907 | Yes |
| 20 | PSTK | na | 26405 | -0.115 | -0.0509 | Yes |
| 21 | DARS2 | na | 27278 | -0.132 | 0.0095 | Yes |
| 22 | MARS2 | na | 32383 | -0.316 | 0.0676 | Yes |
Table: GSEA details [plain text format]

  

Fig 2: KEGG\_AMINOACYL\_TRNA\_BIOSYNTHESIS      
 Blue-Pink O' Gram in the Space of the Analyzed GeneSet

  

Fig 3: KEGG\_AMINOACYL\_TRNA\_BIOSYNTHESIS: Random ES distribution      
 Gene set null distribution of ES for **KEGG\_AMINOACYL\_TRNA\_BIOSYNTHESIS**
